# Development of Au Nanoparticle Two-Dimensional Assemblies Dispersed with Au Nanoparticle-Nanostar Complexes and Surface-Enhanced Raman Scattering Activity

**DOI:** 10.3390/nano14090764

**Published:** 2024-04-26

**Authors:** Kosuke Sugawa, Kaichi Ono, Ritsurai Tomii, Yuka Hori, Yu Aoki, Koki Honma, Kaoru Tamada, Joe Otsuki

**Affiliations:** 1 Department of Materials and Applied Chemistry, College of Science and Technology, Nihon University, Kanda-Surugadai, Chiyoda-ku, Tokyo 101-8308, Japan; 2 Institute for Materials Chemistry and Engineering (IMCE), Kyushu University, 744 Motooka, Nishiku, Fukuoka 819-0395, Japan

**Keywords:** surface-enhanced resonance Raman scattering, two-dimensional assemblies, Au nanostars, self-assembly, heterostructures, localized surface plasmon resonance

## Abstract

We recently found that polyvinylpyrrolidone (PVP)-protected metal nanoparticles dispersed in water/butanol mixture spontaneously float to the air/water interface and form two-dimensional assemblies due to classical surface excess theory and Rayleigh–Bénard–Marangoni convection induced by butanol evaporation. In this study, we found that by leveraging this principle, a unique structure is formed where hetero gold nanospheres (AuNPs)/gold nanostars (AuNSs) complexes are dispersed within AuNP two-dimensional assemblies, obtained from a mixture of polyvinylpyrrolidone-protected AuNPs and AuNSs that interact electrostatically with the AuNPs. These structures were believed to form as a result of AuNPs/AuNSs complexes formed in the water/butanol mixture floating to the air/water interface and being incorporated into the growth of AuNP two-dimensional assemblies. These structures were obtained by optimizing the amount of mixed AuNSs, with excessive addition resulting in the formation of random three-dimensional network structures. The AuNP assemblies dispersed with AuNPs/AuNSs complexes exhibited significantly higher Raman (surface-enhanced resonance Raman scattering: SERRS) activity compared to simple AuNP assemblies, while the three-dimensional network structure did not show significant SERRS activity enhancement. These results demonstrate the excellent SERRS activity of AuNP two-dimensional assemblies dispersed with hetero AuNPs/AuNSs complexes.

## 1. Introduction

Surface-enhanced Raman scattering (SERS) is a highly sensitive molecular fingerprinting technique with applications expected in fields such as diagnostics and biomedical imaging [1,2]. When functional molecules are in close proximity to noble metal nanoparticles, their Raman signals can be significantly enhanced depending on the strong electromagnetic fields generated upon excitation of localized surface plasmon (LSP) resonance of the nanoparticles [3,4,5]. The high-density assembly of noble metal nanoparticles has been recognized as a valuable means to control the properties of local electromagnetic fields. This is because the proximity of multiple plasmonic metal nanoparticles within such assemblies can induce plasmon coupling, potentially generating significantly concentrated electromagnetic fields, known as hotspots, within the nanogap spaces between the particles [6,7,8,9,10]. Therefore, the development of techniques to further control these plasmon coupling properties has led to the development of platforms with even higher SERS activity [11,12].

Typically, the plasmon coupling has been induced by the proximity of identical nanoparticles [6]. In addition, one approach that has recently gained attention for controlling the properties of plasmon coupling is the utilization of the coupling between LSPR resonances of metal nanoparticles of different shapes [13,14,15], sizes [16,17], and materials [18,19,20]. In studies modeling heterodimers composed of spherical gold nanoparticles (AuNPs) and gold nanocubes, variations in the dimer morphology have been reported to result in variations in the plasmon coupling properties, such as resonance wavelength [13] and electromagnetic field intensity [14,21]. Furthermore, gold nanostars (AuNSs), which possess a star-shaped structure, not only induce significant local electromagnetic fields through their LSP resonance [22,23] but also have the potential to exhibit even stronger fields when coupled with the LSP resonance of AuNPs in the AuNPs/AuNSs complexes [24,25]. On the other hand, many of the morphologies used in these studies involve heterodimer structures where two nanoparticles are brought into close proximity [13,14], or core-satellite structures [26]. These structures are suitable for studying the fundamental properties of plasmon coupling, but their application as SERS platforms that utilize the strong electromagnetic field spaces is challenging. Therefore, research focusing on utilizing this distinctive plasmon coupling in high-density two-dimensional assemblies of metal nanoparticles, which have demonstrated utility as SERS sensing platforms [27,28,29], has recently attracted particular attention [30,31]. For example, the SERS activity of two-dimensional assemblies composed of two components, AuNPs and Au@Ag nanocubes, has been reported to be significantly stronger compared to the two-dimensional assemblies consisting of single-component Au nanoparticles or Au@Ag nanocubes alone, attributed to strong plasmon coupling in heterodimers [30]. However, such studies remain rare, and the development of two-dimensional assemblies of metal nanoparticles exhibiting the aforementioned distinctive plasmon coupling properties could be a significant approach towards further SERS-active platforms.

Two-dimensional assemblies of metal nanoparticles have frequently been developed using bottom-up approaches, which offer advantages in terms of cost and simplicity. Many of these techniques utilize the interactions between metal nanoparticles, as well as between nanoparticles and the interface induced by the evaporation of dispersed solvents [32,33] and surface modifiers (or promoters) [34,35], to assemble metal nanoparticles at air/liquid or liquid/liquid interfaces. In addition, we have recently established a method for developing two-dimensional assemblies from metal nanoparticles dispersed in a mixed solution of short-chain alcohol (butanol) and water, based on classical surface excess theory and Rayleigh–Bénard–Marangoni convection induced by the vaporization of butanol [36]. The characteristic of this method is that metal nanoparticles protected by polyvinylpyrrolidone (PVP) interact strongly with butanol in the mixed solution, are induced to the air–liquid interface through microscopic convection, and then assembled. In this study, we observed the spontaneous formation of unique structures, where AuNPs/AuNSs complexes were sparsely dispersed within AuNP assemblies (AuNP/AuNS-in-assemblies), from a water/BuOH solution containing PVP-protected AuNPs and AuNSs interacting electrostatically with them. Here, “AuNPs/AuNSs complexes” refer to composite structures formed from AuNSs and AuNPs through electrostatic interactions between them. Furthermore, notably, the SERS activity of these assemblies was higher compared to simple two-dimensional assemblies of AuNPs. These results suggest that the developed assemblies function as high-performance SERS sensing platforms incorporating the characteristic plasmon coupling between AuNSs and AuNPs.

## 2. Materials and Methods

### 2.1. Materials

In this study, Milli-Q water (18.2 MΩ) was used to prepare all aqueous solutions. Chloroauric(III) acid tetrahydrate (HAuCl_4_, Nacalai Tesque, Kyoto, Japan), trisodium citrate dihydrate (Kanto Chemical, Tokyo, Japan), hydrochloric acid (Kishida Chemical, Osaka, Japan), silver nitrate (Fujifilm Wako Pure Chemical, Osaka, Japan), L-ascorbic acid (Tokyo Chemical Industry, Tokyo, Japan), 1-butanol (BuOH, Kishida Chemical), ethanol (Kishida Chemical), sodium hydroxide (Kanto Chemical), 16-mercaptohexadecanoic acid (MHA, Sigma–Aldrich, St. Louis, MO, USA), polyethyleneimine (PEI, MW: ~10,000, Fujifilm Wako Pure Chemical) and PVP (MW: ~55,000, Sigma–Aldrich, Saint Louis, MO, USA) were used without further purification.

### 2.2. Synthesis of AuNPs and AuNSs

The synthesis of AuNPs was achieved by reducing HAuCl_4_ with citric acid [37]. Specifically, AuNPs were synthesized by refluxing a 0.01 wt.% HAuCl_4_ aqueous solution at 100 °C for 30 min, followed by the addition of a 1 wt.% aqueous solution of trisodium citrate (0.9 mL) and continuing the reflux for an additional 1 h. AuNSs were synthesized using a modified seed-mediated method [38]. The gold nanoparticle seeds were synthesized using the citrate reduction method mentioned above. The volume of a 1 wt.% trisodium citrate solution added to the HAuCl_4_ aqueous solution was adjusted to 4 mL to synthesize the gold nanoparticle seeds with a diameter of 12 nm. A 1 mL colloidal aqueous solution of gold nanoparticle seeds was mixed with 100 mL of 0.01 wt.% HAuCl_4_ aqueous solution, 700 μL of 1 mM hydrochloric acid, and 1 mL of 2 mM silver nitrate. Upon addition of 500 μL of an aqueous solution containing 0.1 M L-ascorbic acid to this mixed solution, AuNSs were synthesized. To prevent aggregation of the AuNSs, 0.7 mL of a 1 wt.% trisodium citrate aqueous solution and 3 mL of a 1 M sodium hydroxide aqueous solution were added, followed by stirring the mixture for approximately 10 min.

### 2.3. Surface Modification of AuNPs and AuNSs

The surface of citrate-modified AuNPs was sequentially modified with MHA, PEI, and PVP. A 5 mM MHA ethanol solution (0.9 mL) was added to the colloidal AuNPs solution (15 mL) under stirring, followed by stirring for approximately 15 min to immobilize MHA on the AuNPs surface. The resulting colloidal solution underwent purification through centrifugation (5600 rpm, 5 min). The resulting precipitate was dispersed in 5 mL of ethanol and added to a 15 mL ethanol solution of 4 mg/mL PEI, followed by stirring for 15 min to electrostatically immobilize PEI onto the MHA-modified AuNPs. This solution was also purified by centrifugation (6500 rpm, 10 min) and dispersed in 5 mL of ethanol. The colloidal solution was then added to a 0.7 mM ethanol solution of PVP (15 mL) and stirred for approximately 15 min to modify the outermost surface of the AuNPs with PVP [36]. This mixed solution underwent centrifugation twice (7000 rpm, 10 min) and was finally dispersed in BuOH (0.12 mL). The surface of AuNSs was modified with MHA utilizing the same method as the MHA modification on the surface of AuNPs.

### 2.4. Self-Assembly of Au Nanoparticles at the Air/Water Interface

A solution of BuOH (0.12 mL) containing 5.5 × 10^11^ AuNPs with PVP-modified surfaces was mixed with an aqueous solution (2.4 mL) containing 5.3 × 10^8^, 1.1 × 10^9^, 2.1 × 10^9^, 3.2 × 10^9^, or 4.3 × 10^9^ AuNSs with MHA-modified surfaces in a sample vial and stirred for 10 s. Afterwards, the mixed solution was quickly transferred to a petri dish with a diameter of 2.8 cm and left to stand. For all samples, a liquid-like film of nanoparticles formed at the air/water interface within 90 min. The nanoparticle assemblies, which were transferred on the surface of glass plate, were treated with low-pressure plasma (intensity: 10, time: 90 s).

### 2.5. Measurements

UV-visible near-infrared spectroscopy was performed using a JASCO V-770 spectrophotometer (Tokyo, Japan). Field-emission scanning electron microscopy (FE-SEM) images were taken using a Hitachi S-4500. (Tokyo, Japan) Transmission electron microscopy (TEM) images were taken using a Hitachi HF-2000 (Tokyo, Japan). Atomic force microscopy (AFM) measurements were performed with a Hitachi SPI-3800N-SPA400 (Tokyo, Japan). The Raman scattering spectra were measured using a micro-Raman spectrometer (JASCO NRS-4100, Tokyo, Japan). A 785 nm continuous-wave (CW) laser (Sun Instruments, Tokyo, Japan) was used as the excitation light source. The modification of the probe molecule, indocyanine green (ICG), onto the sample substrate was achieved by casting a 50 μL solution of 1 × 10^−^^6^ M ICG onto the sample substrate and allowing it to dry. The laser was directed onto the sample surface through a 20× working distance objective lens. Dynamic light scattering measurements were performed using a Zetasizer Nano ZS (Malvern Panalytical, Great Malvern, UK).

## 3. Results

### 3.1. Construction of AuNPs/AuNSs Complex-Dispersed AuNP Two-Dimensional Assemblies

**Scheme 1:**
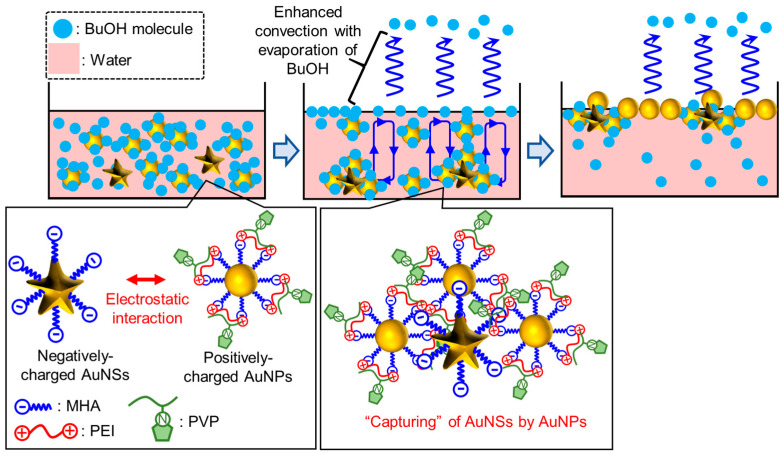
Scheme for the fabrication of AuNP/AuNS-in-assemblies.

In this study, the estimated formation mechanism of AuNP/AuNS-in-assemblies is illustrated in Scheme 1. The AuNSs are assumed to be modified with MHA, while the AuNPs are assumed to be sequentially modified with MHA, PEI, and PVP. As a result, the surface of AuNSs is expected to carry a negative charge, while that of AuNPs is expected to be positively charged, reflecting the charges of the modified molecules. Furthermore, we have previously confirmed that only the metal nanoparticles protected by PVP, which strongly interact with BuOH, assemble at the air/water interface using the assembly method we developed in the past [36]. Therefore, AuNSs dispersed in water/BuOH are not expected to spontaneously assemble at the air/water interface. On the other hand, the coexistence of AuNPs and AuNSs in a water/BuOH solution is expected to lead to the formation of complexes between them through electrostatic attraction within the solution. As a result, AuNPs/AuNSs complexes, composed of AuNPs capable of assembling at the air/water interface, are expected to accumulate at the interface. On the other hand, if the number of AuNPs significantly exceeds that of AuNSs, it is anticipated that AuNPs, which do not form complexes with AuNSs, will independently assemble at the air/water interface, resulting in the formation of high-density two-dimensional assemblies. Consequently, the formation of AuNP/AuNS-in-assemblies is anticipated.

In the experiments, AuNPs with an average diameter of 53 ± 5 nm and AuNSs with sizes ranging approximately from 130 to 200 nm were used (TEM image: Figure S1). As anticipated, zeta potential measurements revealed surface charges of −42.4 mV for AuNSs modified with MHA and 8.16 mV for AuNPs modified with MHA-PEI-PVP. These nanoparticles were dispersed in a water/BuOH solution. The number of AuNPs in the solution was fixed at 5.5 × 10^11^, while the number of AuNSs was varied in five increments ranging from 5.3 × 10^8^ to 4.3 × 10^9^.

**Figure 1:**
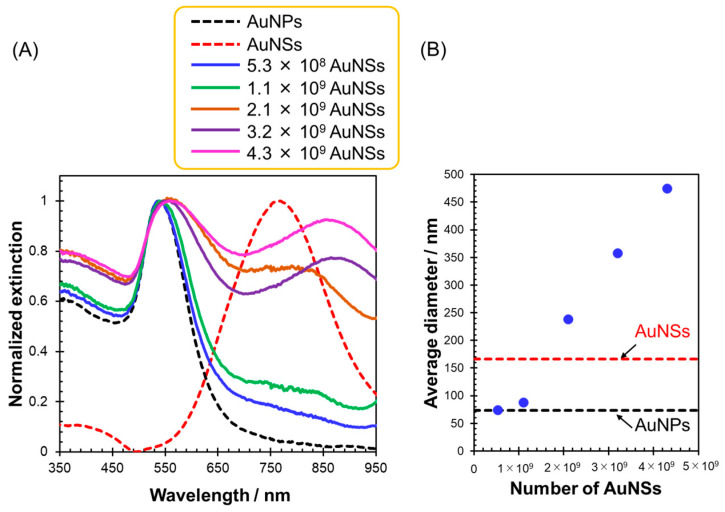
Verification of the formation of AuNPs/AuNSs complexes. (**A**) Extinction spectra of the colloidal solution obtained immediately after mixing AuNPs with AuNSs in water/BuOH. (**B**) Plots of average hydrodynamic diameter of the colloidal solution estimated by DLS measurements immediately after mixing AuNPs with AuNSs in water/BuOH.

First, to verify the formation of AuNPs/AuNSs complexes in water/BuOH, the extinction spectrum of the colloidal solution immediately after mixing both nanoparticles in the solution was measured (Figure 1A). Under conditions where the number of AuNSs was high (>1.1 × 10^9^), a distinct LSP resonance band of AuNSs was observed in the near-infrared region, in addition to that of AuNPs in the visible range. The resonance wavelength of AuNSs shifted to longer wavelengths compared to that of AuNSs dispersed in water/BuOH solution (without AuNPs) used as a reference. This resonance wavelength shift is similar to that observed in the formation of AuNSs (core)/AuNPs (satellite) nanostructures reported in the past [24]. Therefore, this resonance wavelength shift is attributed to the plasmon coupling between AuNSs and AuNPs, suggesting the formation of AuNPs/AuNSs complexes. The LSP resonance of AuNSs was not clearly observed under conditions where the number of AuNSs was low (<2.1 × 10^9^); however, the formation of AuNSs/AuNPs complexes is still anticipated. Furthermore, the average hydrodynamic diameter of the colloidal solution immediately after mixing both nanoparticles in water/BuOH was measured by DLS measurements (Figure 1B). Under conditions where the number of AuNSs was low (5.3 × 10^8^ and 1.1 × 10^9^), the particles exhibited a hydrodynamic diameter similar to that of AuNPs measured as a reference (74 nm) in BuOH. However, as the number of AuNSs in the solution increased, the hydrodynamic diameter also increased, surpassing both the hydrodynamic diameters of AuNPs and AuNSs (166 nm). These results suggest the formation of larger nanoparticles (i.e., AuNSs/AuNPs complexes) in water/BuOH compared to AuNSs alone. When the number of AuNSs was low, it is likely that there was not a significant change in hydrodynamic diameter, as the number of AuNSs/AuNPs complexes was overwhelmingly fewer than the number of AuNPs. From these results, it is suggested that when AuNSs and AuNPs were mixed in water/BuOH, their complexes were formed immediately.

**Figure 2:**
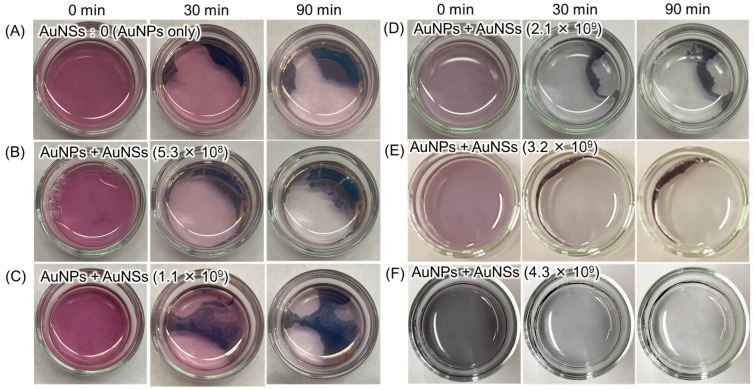
Photographs of the assembling process of nanoparticles from water/BuOH to air/water interface. (**A**) The formation of assemblies from the colloidal solution of AuNPs (quantity: 5.5 × 10^11^) without AuNSs. (**B**–**F**) The assemblies formed from solutions where AuNPs (quantity: 5.5 × 10^11^) were mixed with (**B**) 5.3 × 10^8^, (**C**) 1.1 × 10^9^, (**D**) 2.1 × 10^9^, (**E**) 3.2 × 10^9^, and (**F**) 4.3 × 10^9^ AuNSs, respectively.

Next, the assembling behavior of these nanoparticles to the air/water interface was investigated. When only AuNSs were dispersed in the water/BuOH solution, there was no assembling of AuNSs to the air/water interface even after 5 h. This is because, as mentioned above, the assembling properties to the air/water interface are only exhibited by the nanoparticles protected with PVP [36]. Therefore, when only PVP-protected AuNPs were dispersed in water/BuOH, the red color attributed to the LSP resonance of AuNPs in the solution faded with time, and a uniform blue liquid-like film formed at the air/water interface (Figure 2A). This is the result of the spontaneous assembly of AuNPs dispersed in water/BuOH at the air/water interface. Next, the color of the mixed solution of AuNPs and AuNSs is discussed. Under conditions of low AuNS numbers (Figure 2B,C), the solution color immediately after mixing AuNPs with AuNSs was similar to that in Figure 2A. This is supported by the fact that, as shown in Figure 1A, the LSP resonance band of AuNPs remained largely unchanged, while the LSP resonance band of AuNSs is significantly lower compared to that of AuNPs, due to the low number of mixed AuNSs. On the other hand, in Figure 2D–F, the color of the solution faded from red and exhibited a darker blue hue compared to the AuNPs colloidal solution. This corresponds to significant spectral changes in the extinction spectrum (i.e., broadening of the LSP resonance band of AuNPs and enhancement of the LSP resonance band of AuNSs, as shown in Figure 1A), resulting from the increase in the number of AuNSs and the formation of numerous AuNPs/AuNSs complexes. Furthermore, it is worth emphasizing that even when various numbers of AuNSs were mixed with AuNPs in water/BuOH, the solution color faded with time, and the formation of a distinct liquid-like film on the air/water interface was observed (Figure 2B–F). After 90 min, the solution color became almost colorless and transparent for all samples, suggesting that both AuNPs alone and AuNPs/AuNSs complexes accumulated from the solution to the air/water interface. It is worth noting that as the number of AuNSs increased, the color of the film formed at the air/water interface became darker, and the area of the film decreased. Particularly, in samples with AuNS numbers of 3.2 × 10^9^ and 4.3 × 10^9^, darker films were observed only at the rim of the dish. The reason for this position-selective accumulation is currently unclear. Furthermore, the extinction spectra of the water/BuOH solutions accompanying the nanoparticle assembly at the air/water interface were measured.

**Figure 3:**
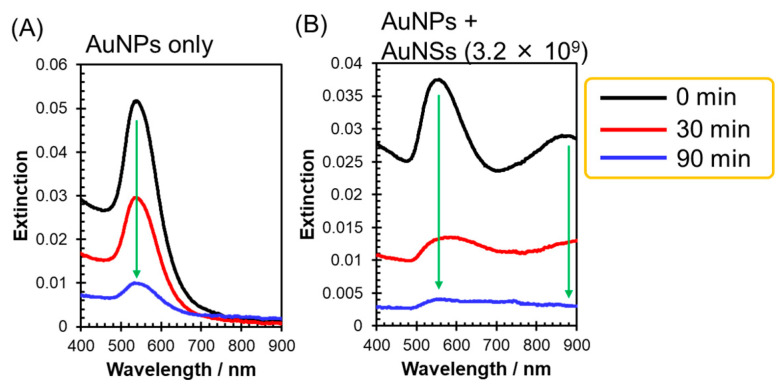
Time-dependent extinction spectra of the water/BuOH solution: (**A**) Only dispersed AuNPs (quantity: 5.5 × 10^11^) and (**B**) AuNPs (quantity: 5.5 × 10^11^) with AuNSs (quantity: 3.2 × 10^9^).

The spectrum of the water/BuOH solution containing only dispersed AuNPs, measured for reference (Figure 3A), showed a decrease in the LSP resonance band of AuNPs in the visible range with time. This decrease is attributed to the assembly of AuNPs to the air/water interface, leading to a decrease in the number of AuNPs in the solution. Additionally, Figure 3B shows the extinction spectrum of a solution where both AuNPs and 3.2 × 10^9^ AuNSs are mixed together as a typical example (the extinction spectra for conditions with other numbers of AuNSs are presented in Figure S2). In addition to the decrease in the LSP resonance band of AuNPs with time, the extinction band in the near-infrared region attributed to the LSP resonance band of the AuNPs/AuNSs complexes also decreased similarly to that of AuNPs. These results confirm that AuNSs alone do not accumulate at the air/water interface; however, after forming complexes with AuNPs, they do assemble at the air/water interface. When AuNPs form complexes with AuNSs, it suggests that the capability of AuNPs to accumulate at the air/water interface remains intact even within the complexes.

**Figure 4:**
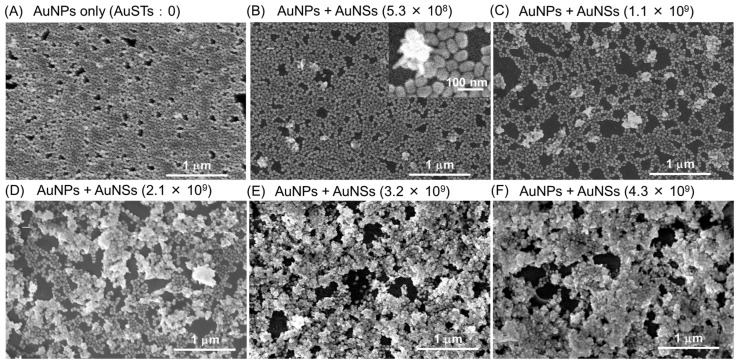
SEM images of the assemblies. (**A**) SEM image of assemblies consisting solely of AuNPs obtained from the colloidal solution of AuNPs (without AuNSs). SEM images of AuNP/AuNS-in-assemblies prepared from solutions where AuNPs (quantity: 5.5 × 10^11^) were mixed with (**B**) 5.3 × 10^8^, (**C**) 1.1 × 10^9^, (**D**) 2.1 × 10^9^, (**E**) 3.2 × 10^9^, and (**F**) 4.3 × 10^9^ AuNSs.

Next, the nanoparticle assemblies formed at the air/water interface were transferred onto glass substrates, and their morphology was observed using SEM. As shown in Figure 4A, the assemblies formed solely with AuNPs exhibited high-density two-dimensional structures. On the other hand, when the number of AuNSs was 5.3 × 10^8^ and 1.1 × 10^9^ (Figure 4B,C), typical AuNP/AuNS-in-assemblies were formed (inset in Figure 4B), with AuNPs/AuNSs complexes sparsely dispersed throughout the AuNP two-dimensional assemblies. When the number of AuNSs was >2.1 × 10^9^, the two-dimensional assemblies of AuNPs was hardly observed, and only a heterogeneous three-dimensional network structures consisting of AuNPs and AuNSs was observed (Figure 4D–F). This formation is likely attributed to the increase in the number of AuNSs in water/BuOH. Consequently, there is a higher number of AuNPs/AuNSs complexes, which then aggregated randomly at the air/water interface due to strong hydrophobic interactions among these complexes.

**Figure 5:**
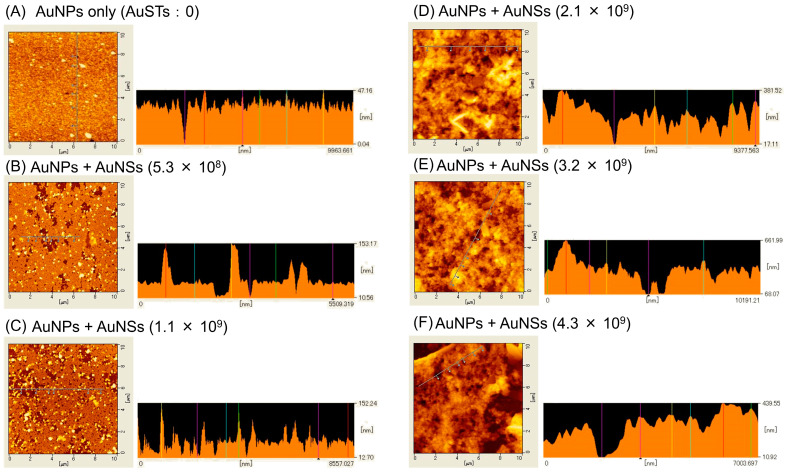
AFM images of the assemblies. (**A**) AFM image of assemblies consisting solely of AuNPs obtained from the colloidal solution of AuNPs (without AuNSs). AFM images of AuNP/AuNS-in-assemblies prepared from solutions where AuNPs (quantity: 5.5 × 10^11^) were mixed with (**B**) 5.3 × 10^8^, (**C**) 1.1 × 10^9^, (**D**) 2.1 × 10^9^, (**E**) 3.2 × 10^9^, and (**F**) 4.3 × 10^9^ AuNSs.

The morphology of these nanoparticle assemblies transferred onto glass substrates was also examined using AFM measurements. In the AuNP assemblies composed solely of AuNPs (Figure 5A), the formation of a uniform nanoparticle film, consistent with the SEM observations (Figure 4A), was confirmed. The average film thickness was measured to be 45 ± 1.8 nm, which was consistent with the size of AuNPs estimated from TEM images (Figure S1). These results indicate the formation of a monolayer of AuNPs. For samples with AuNS numbers of 5.3 × 10^8^ and 1.1 × 10^9^ (Figure 5B,C), larger materials than AuNPs and AuNSs (Figure S3), presumably AuNPs/AuNSs complexes, were observed to be dispersed within the uniform AuNP assemblies. Furthermore, TEM observations (Figure S1C) confirmed that this complex is composed of AuNPs and AuNSs. Additionally, an increase in the number of AuNSs was found to increase the density of AuNPs/AuNSs complexes: when the number of AuNSs was 5.3 × 10^8^, the density of AuNPs/AuNSs complexes was 12 ± 3.3/µm^2^, and when it was 1.1 × 10^9^, the density was 22 ± 2.9/µm^2^. On the other hand, when the number of AuNSs exceeded 2.1 × 10^9^, the formation of a thick (>350 nm) and heterogeneous three-dimensional network structure was observed, consistent with the SEM observations (Figure 5D–F).

### 3.2. Optical Properties and SERS Activity of Assemblies

**Figure 6:**
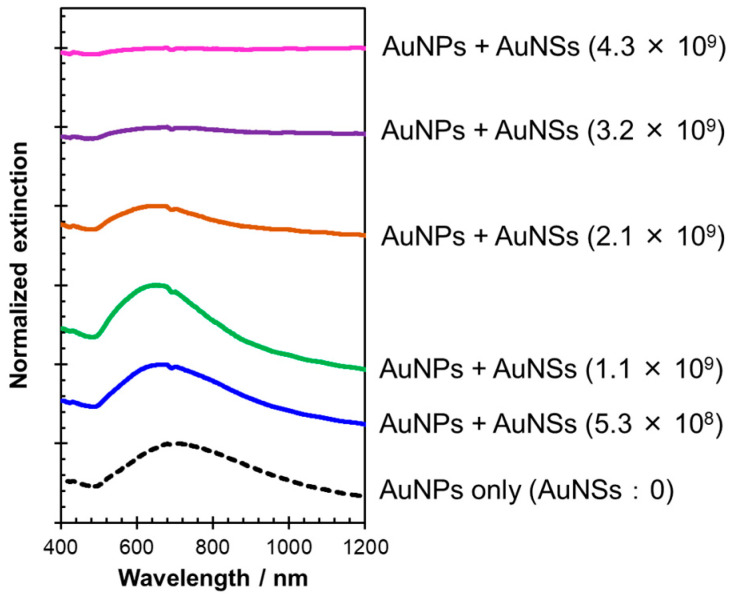
Extinction spectra of AuNP/AuNS-in-assemblies on glass plates obtained from a colloidal solution where AuNPs (quantity: 5.5 × 10^11^) were mixed with 5.3 × 10^8^, 1.1 × 10^9^, 2.1 × 10^9^, 3.2 × 10^9^, and 4.3 × 10^9^ AuNSs. As a reference, the extinction spectrum of assemblies consisting solely of AuNPs obtained from the colloidal solution of AuNPs (without AuNSs) was also provided.

The optical properties of the assemblies produced from water/BuOH containing AuNSs and AuNPs were investigated by measuring their extinction spectra (Figure 6). The assemblies constructed solely with AuNPs as reference samples (i.e., AuNS: 0) exhibited a broad LSP resonance band from 500 to 1000 nm, induced by plasmon coupling between the AuNPs. This optical property was maintained when the number of AuNSs was low (5.3 × 10^8^ and 1.1 × 10^9^). The assemblies formed under these conditions exhibited optical properties dominated by the assemblies of AuNPs rather than the optical properties of AuNPs/AuNSs complexes, as suggested by the low density of AuNPs/AuNSs complexes, as shown in Figure 4 and Figure 5. On the other hand, under conditions where the number of AuNSs exceeded 1.1 × 10^9^, an increase in the number of AuNSs led to significant broadening of the LSP resonance band. This is attributed to the change in the assembly structure to a random three-dimensional network structure, as can be seen from Figure 4 and Figure 5.

**Figure 7:**
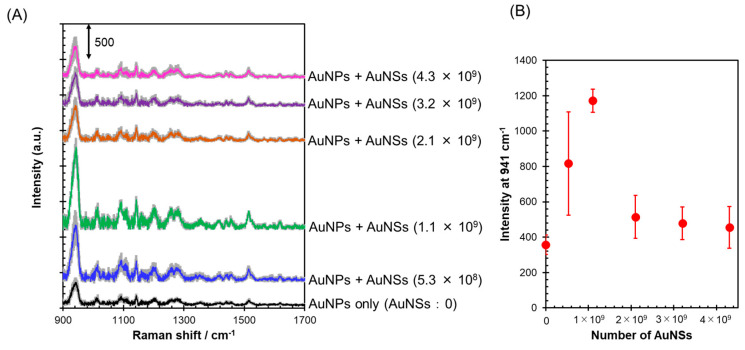
SERRS properties of AuNP/AuNS-in-assemblies. (**A**) SERRS spectra of AuNP/AuNS-in-assemblies modified with ICG on glass plates obtained from a colloidal solution where AuNPs (quantity: 5.5 × 10^11^) were mixed with 5.3 × 10^8^, 1.1 × 10^9^, 2.1 × 10^9^, 3.2 × 10^9^, and 4.3 × 10^9^ AuNSs. As a reference, SERRS spectrum of assemblies consisting solely of AuNPs obtained from the colloidal solution of AuNPs (without AuNSs) was also provided. (**B**) The intensity of SERRS peak against the number of AuNSs.

The SERRS activity of these nanoparticle assemblies was investigated using ICG as a Raman probe. SERRS spectrum patterns derived from ICG were obtained from ICG-modified AuNP/AuNS-in-assemblies, as well as from ICG-modified referenced AuNP two-dimensional assemblies (without AuNSs) (Figure 7A) [32,39,40]. The detailed assignment is described in Figure S4. The SERRS signal from AuNP/AuNS-in-assemblies prepared by mixing with 5.3 × 10^8^ and 1.1 × 10^9^ AuNSs was significantly higher than that from the reference AuNP assemblies (Figure 7B). Additionally, the signal intensity increased with an increase in the number of AuNSs. However, under conditions where the number of AuNSs corresponding to the formation of three-dimensional random network structures exceeded 1.1 × 10^9^, the SERRS signal intensity remained almost unchanged regardless of the number of AuNSs, similar to AuNP assemblies. These results demonstrate that the two-dimensional assemblies composed of AuNPs and AuNPs/AuNSs complexes serve as high-performance SERS platforms.

We examined the resilience of AuNP/AuNS-in-assemblies, developed in this study for potential application as SERS platforms, against various solvents. The assemblies, transferred onto glass substrates beforehand, were thoroughly washed with toluene, Milli-Q water, and ethanol, yet their optical properties remained unchanged (Figure S5A). Furthermore, from photographs of the assemblies (Figure S5B), it is evident that they remained stable without peeling off during the washing process. Therefore, it can be concluded that the robustness of the assemblies as SERS platforms is high.

## 4. Conclusions

In this study, we developed a unique structure where hetero AuNPs/AuNSs complexes were dispersed within AuNP two-dimensional assemblies. This was achieved by mixing AuNPs with AuNSs, which interact electrostatically, during the process of preparing AuNP two-dimensional assemblies using Rayleigh–Bénard–Marangoni convection. The SERRS activity of the structure was found to be higher than that of simple AuNP assemblies and correlated with the density of AuNPs/AuNSs complexes. Therefore, it was demonstrated that low-density AuNPs/AuNSs complexes enhanced the SERRS activity of AuNP two-dimensional assemblies. High-density assemblies of plasmonic metal nanoparticles are expected to be applied as SERS platforms enabling the detection of low-concentration biomolecules and pesticide residues for diagnostics and food safety assurance. Therefore, the AuNP/AuNS-in-assemblies have the potential for applications as SERS platforms enabling more sensitive detection of the aforementioned target molecules. In the future, by developing techniques to incorporate further optimized heterocomplexes with nanoparticles of different shapes, sizes, and materials into metal nanoparticle assemblies, a highly sensitive SERS activity platform can be developed. This strategy is currently being pursued in our laboratory.

## Data Availability

The data supporting the findings of this study are available in the article and its Supplementary Materials.

## Supplementary Materials

The following supporting information can be downloaded at: https://www.mdpi.com/article/10.3390/nano14090764/s1, Figure S1: TEM images of AuNPs and AuNSs. Figure S2: Time-dependent extinction spectra of the water/BuOH solution containing AuNPs and AuNSs. Figure S3: Comparison of morphologies through AFM observations between individual AuNPs and AuNSs, and AuNP/AuNS-in-assemblies Figure S4: Peak assignments of SERRS spectrum of ICG-modified AuNP/AuNS-in-assemblies. Figure S5: Chemical robustness (stability against solvents) of the AuNP/AuNS-in-assemblies. Ref. [41] is cited in Supplementary Materials.
