# Supplementary material for: Development of Au Nanoparticle Two-Dimensional Assemblies Dispersed with Au Nanoparticle-Nanostar Complexes and Surface-Enhanced Raman Scattering Activity
Source: Nanomaterials (Basel). 2024 Apr 26;14(9):764. doi: 10.3390/nano14090764 (PMC11085563; doi:10.3390/nano14090764)
Supplement: Supplementary file 1 [file nanomaterials-14-00764-s001.zip › nanomaterials-2962038-supplementary.pdf]

Development of Structures with Complexes of Au Nanoparticles and Au Nanostars Dispersed within Au Nanoparticle Two-Dimensional Assemblies and Surface-Enhanced Raman Scattering Activity

Kosuke Sugawa[1,*], Kaichi Ono[1], Ritsurai Tomii[1], Yuka Hori[1], Yu Aoki[1], Koki Honma[1], Kaoru Tamada[2], Joe Otsuki[1]

[1]Department of Materials and Applied Chemistry, College of Science and Technology, Nihon University, Kanda-Surugadai, Chiyoda-ku, Tokyo 101-8308, Japan
[2]Institute for Materials Chemistry and Engineering (IMCE), Kyushu University, 744 Motooka, Nishiku, Fukuoka 819-0395, Japan
*Correspondence: sugawa.kosuke@nihon-u.ac.jp

1. Morphologies of AuNPs, AuNSs, and AuNPs/AuNSs complexes

2. Time-dependent extinction spectra of the water/BuOH solution

3. Comparison of morphologies through AFM observations between individual AuNPs and AuNSs, and AuNP/AuNS-in-assemblies

4. Peak assignments of SERRS spectrum of ICG-modified AuNP/AuNS-in-assemblies

5. Chemical robustness (stability against solvents) of the AuNP/AuNS-in-assemblies

1. Morphologies of AuNPs, AuNSs, and AuNPs/AuNSs complexes

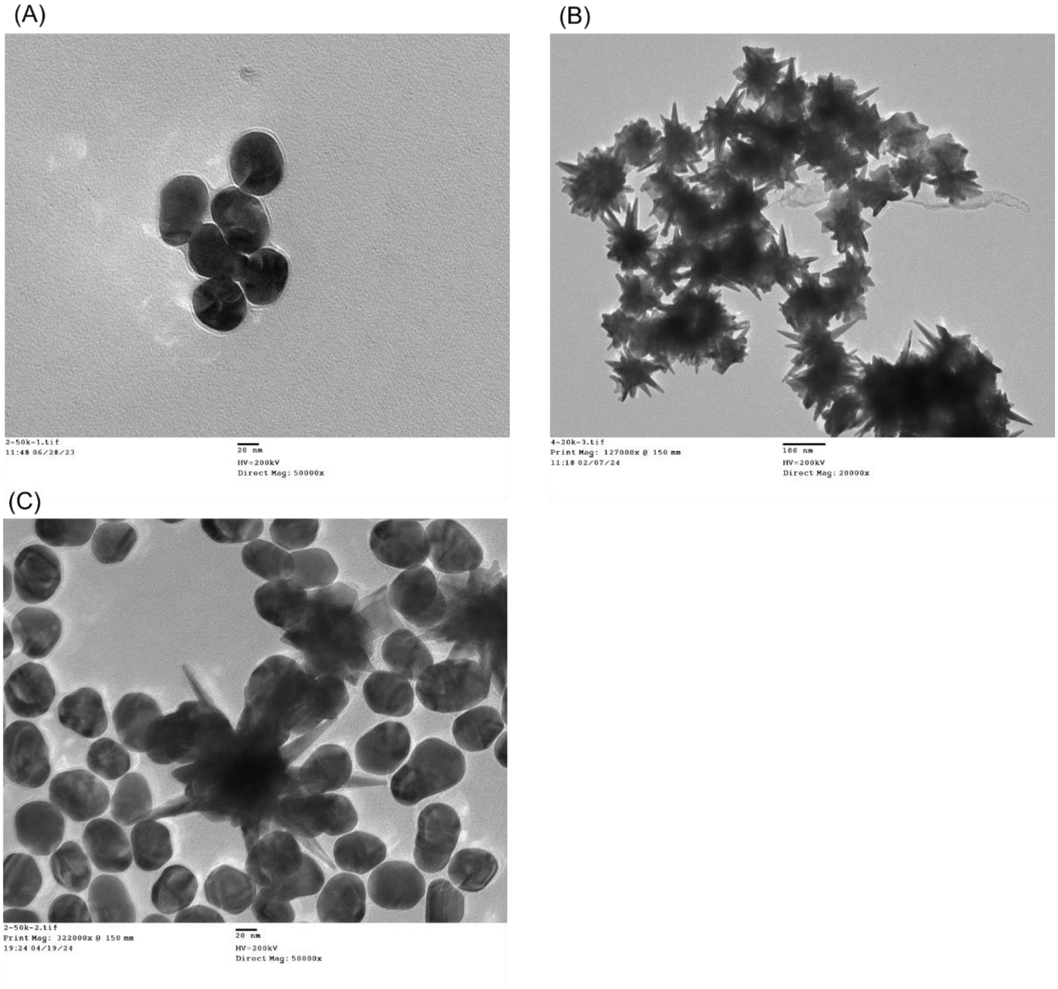

Figure S1. TEM images of (A) AuNPs, (B) AuNSs, and (C) AuNPs/AuNSs complexes.

2. Time-dependent extinction spectra of the water/BuOH solution

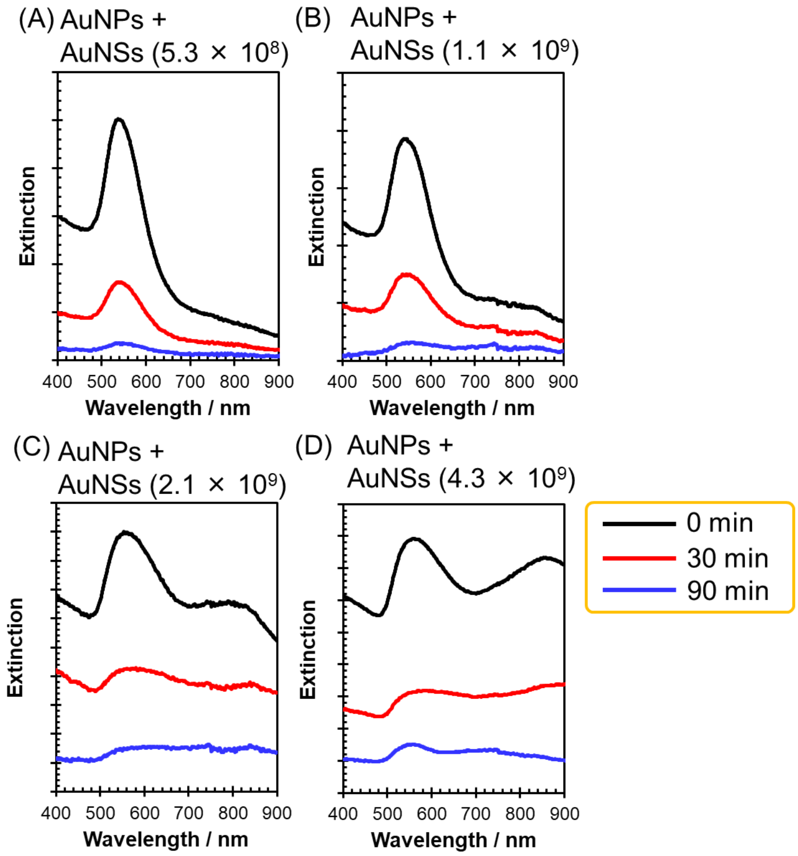

Figure S2. Time-dependent extinction spectra of the water/BuOH solution containing AuNSs (quantity: 5.5 × 1011) and AuSTs (quantity: (A) 5.3 × 10^8, (B) 1.1 × 10^8, (C) 2.1 × 10^9, (D) 4.3 × 10^9).

3. Comparison of morphologies through AFM observations between individual AuNPs and AuNSs, and AuNP/AuNS-in-assemblies

The dispersed AuNPs and AuNSs immobilized on quartz substrates (**Figure S3**(A) and (B)) were prepared utilizing electrostatic interactions between the quartz substrate modified with polymer electrolytes and these nanoparticles, following previous reports.[S1] From the cross-sectional analysis of these nanoparticles, the average height profiles of AuNPs and AuNSs were determined to be 43 ± 3.2 nm and 95 ± 9.0 nm, respectively. It was observed that the average height profiles of the dispersed materials in AuNPs assemblies (**Figure S3**(C): 143 ± 4.6 nm and (D): 152 ± 12 nm) were significantly larger than these values.

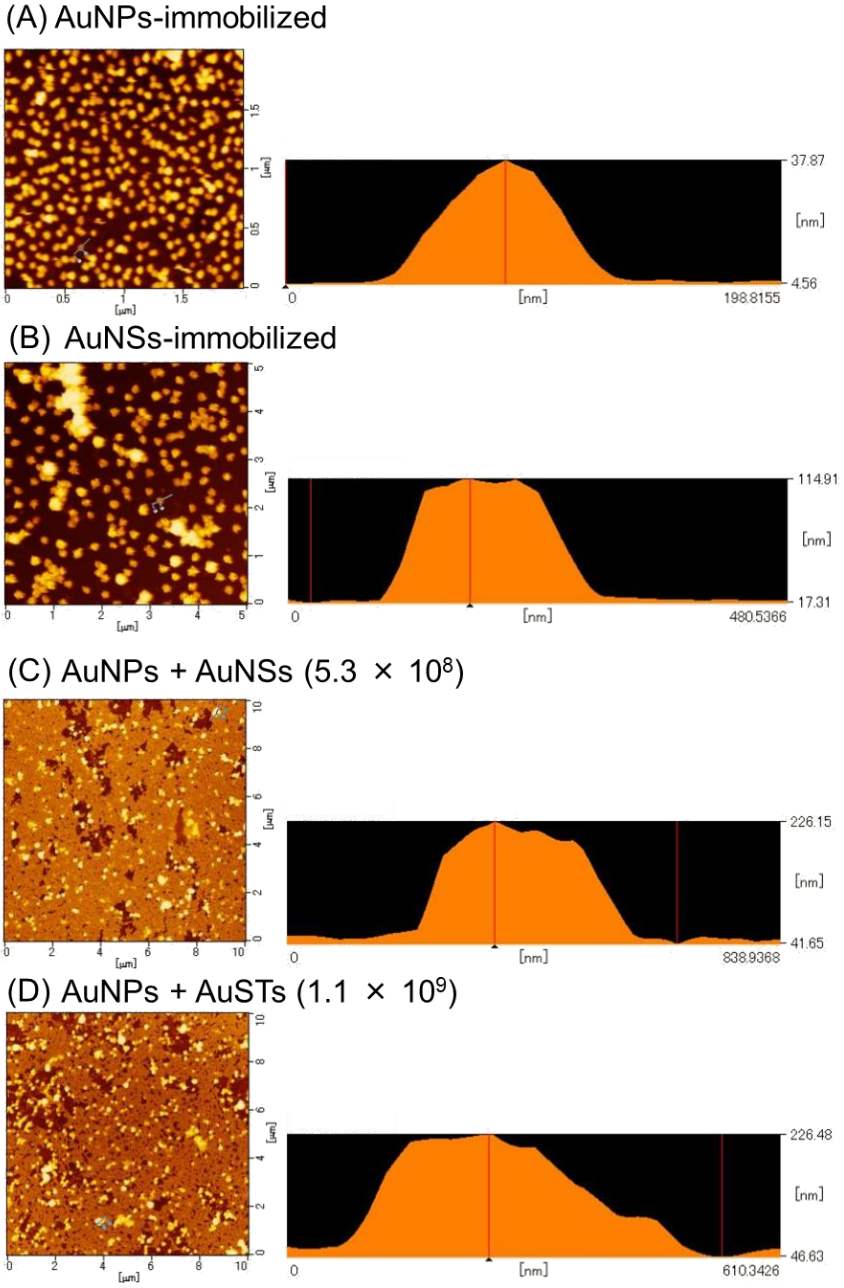

Figure S3. AFM images and cross-sectional analyses for (A) AuNPs-immobilized quartz substrate, (B) AuNSs-immobilized quartz substrate, (C) AuNPs + AuNSs(5.3 × 10^8), and (D) AuNPs + AuNSs(1.1 × 10^9).

4. Peak assignments of SERRS spectrum of ICG-modified AuNP/AuNS-in-assemblies

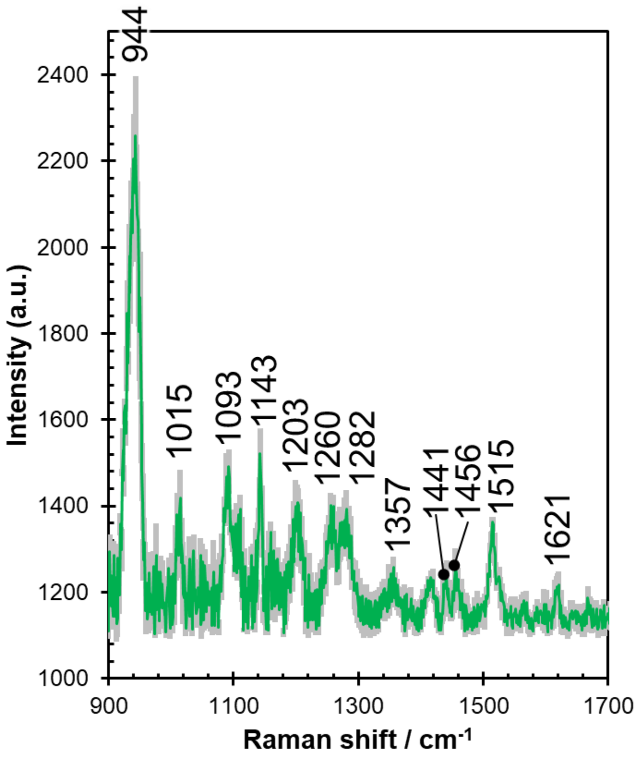

Peak position / cm^{-1}	Assignments
944	γ(CH)
1015	ν(CC)
1093	ν(SO_3^-)
1143	ν(phenyl ring), δ(CH)
1203	δ(CH)
1260	δ(CH)
1282	δ(CH)
1357	ν(pyrrole rings), δ(CH)
1441	δ(CH)
1456	δ(CH)
1515	δ(CH)
1621	ν(C=C)

Figure S4. Peak assignments of SERRS spectrum of ICG-modified AuNP/AuNS-in-assemblies, which were obtained from a colloidal solution containing AuNPs and 1.1 × 10^9 AuNSs.

5. Chemical robustness (stability against solvents) of the AuNP/AuNS-in-assemblies

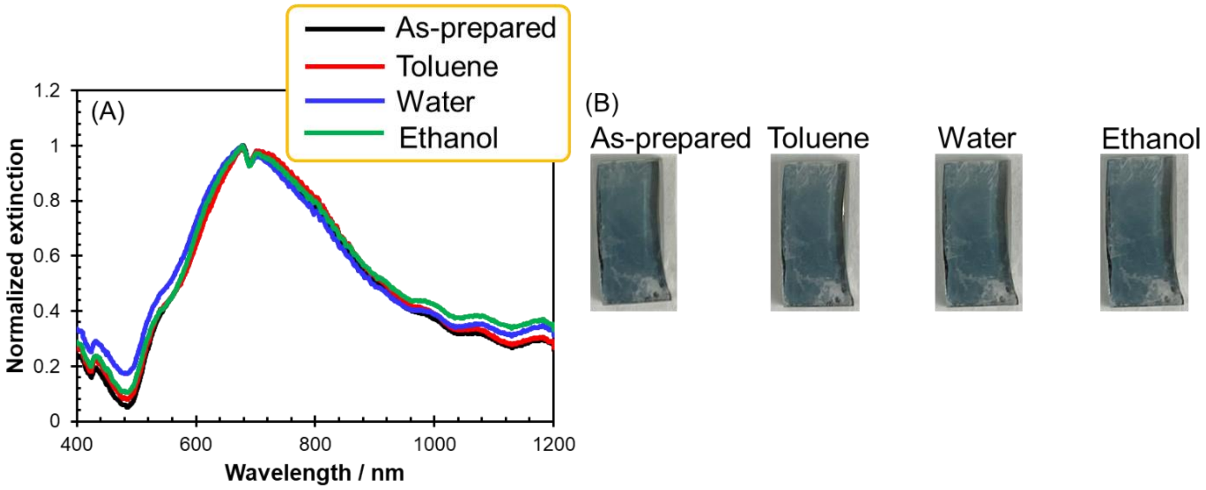

Figure S5. (A) Extinction spectra of AuNP/AuNS-in-assemblies obtained from a colloidal solution containing 5.5×10^{11} AuNPs and 1.1×10^{9} AuNSs. The spectra are shown for the assemblies immediately after preparation (as-prepared) and after washing with toluene, water, and ethanol. (B) The picture images show the assemblies immediately after preparation (as-prepared) and after washed with toluene, water, and ethanol.

References

S1. Sugawa, K.; Takeshima, N.; Uchida, K.; Tahara, H.; Jin, S.; Tsunenari, N.; Akiyama, T.; Kusaka, Y.; Fukuda, N.; Ushijima, H.; Tsuchido, Y.; Hashimoto, T.; Hayashita, T.; Otsuki, J. Photocurrent enhancement of porphyrin molecules over a wide-wavelength region based on combined use of silver nanoprisms with different aspect ratios. *J. Mater. Chem. C* **2015**, *3*, 11439-11448. doi: 10.1039/C5TC02606G.
